# Biomarkers of systemic treatment response: MR images of intratumoral fat deposition in colorectal liver metastases (CRLM) after chemotherapy

**DOI:** 10.1007/s00384-024-04762-0

**Published:** 2024-11-20

**Authors:** Irmina Morawska, Katarzyna Pasicz, Andrzej Cieszanowski

**Affiliations:** 1 Department of Radiology I, The Maria Sklodowska-Curie National Research Institute of Oncology, 5 Wilhelm Conrad Roentgen St, 02-781 Warsaw, Poland; 2 Department of Medical Physics, The Maria Sklodowska-Curie National Research Institute of Oncology, 5 Wilhelm Conrad Roentgen St, 02-781 Warsaw, Poland; 3 Department of Clinical Radiology II, Faculty of Medicine, Medical University of Warsaw, 1A Stefan Banach St, 02-097 Warsaw, Poland

**Keywords:** Intratumoral fat deposition, Signal intensity index, Chemical shift MRI, DIXON technique, Colorectal liver metastases, Chemotherapy

## Abstract

**Purpose:**

Colorectal cancer cells containing mobile lipids are said to be an early indicator of chemotherapy effects. The objective of the study was to examine the frequency and clinical relevance of intratumoral fat deposition in colorectal liver metastases (CRLM) post-chemotherapy using dual-echo chemical shift gradient-echo magnetic resonance imaging (MRI).

**Methods:**

A retrospective analysis of 98 patients with CRLM diagnosed between 2017 and 2022 (69 M, mean age 62.87 ± 10.73 years old) who had an MRI after chemotherapy was performed. On dual-echo chemical shift gradient-echo MRI, intratumoral fat deposition of CRLM was evaluated. A signal intensity drop of ≥ 12% in opposed-phase images vs. in-phase images indicated intratumoral fat. After chemotherapy, the presence of fat deposition was correlated with patients’ overall survival.

**Results:**

Before and after chemotherapy, 0 (0%) and 29 (29.59%) patients exhibited intratumoral fat. The number of CRLM ranged from 1 to 25 with a median of 3 and a mean size of 32.58 ± 22.95 mm. The groups had statistically different survival times. Overall survival was shorter for patients with intratumoral fat deposition in CRLM (32 months (24–60, 95% CI)) than for patients without fat deposition in CRLM (48 months (36–NA, 95% CI)).

**Conclusion:**

In our group, nearly 30% of CRLM patients exhibited intratumoral fat after chemotherapy. Patients with intratumoral fat deposition in CRLM have a shorter overall survival time. The presence of fat in CRLM correlates with a poor long-term prognosis.

## Introduction

Colorectal liver metastases (CRLM) represent a significant clinical challenge, as the liver is a common site for metastasis in colorectal cancer (CRC), complicating patient management and prognosis. Neoadjuvant chemotherapy is a cornerstone of CRLM treatment, aimed at reducing tumor burden and enhancing the prospects of successful surgical resection [1, 2]. Traditionally, treatment response has been evaluated using radiological criteria, such as the Response Evaluation Criteria in Solid Tumors (RECIST) version 1.1, which primarily focuses on changes in tumor size [3]. However, recent studies have highlighted the need for a more nuanced understanding of the effects of chemotherapy beyond size reduction, including its impact on the tumor microenvironment, particularly with regard to intratumoral fat deposition.

Several chemotherapeutic agents, including irinotecan and oxaliplatin, have been associated with inducing fat accumulation within CRLM [4]. The extent and clinical significance of this fat accumulation appear to be influenced by a variety of factors, including chemotherapy type and duration, as well as patient-specific factors such as metabolic comorbidities and genetic predispositions. This has led to a growing interest in understanding how intratumoral fat deposition affects tumor behavior and clinical outcomes.

For example, Wu et al. demonstrated that lipid-rich cancer cells, including those from colorectal tumors, may contain mobile lipids that serve as early indicators of chemotherapy-induced stress, potentially contributing to tumor cell cytotoxicity [5]. Conversely, Vishwa et al. proposed that chemotherapy-induced lipid storage within tumors could modify the tumor microenvironment, potentially facilitating tumor growth and resistance to therapy [6]. In contrast, Alkafaas et al. suggested that chemotherapy might reduce lipid levels by disrupting lipid synthesis and promoting degradation, which could weaken the tumor and enhance treatment efficacy [7].

Despite these divergent findings, the clinical significance of intratumoral fat remains unclear, particularly in terms of its prognostic implications. Nakai et al., in a cohort of 59 patients, observed that CRLM with intratumoral fat was associated with shorter overall survival compared to CRLM without fat [8]. This finding highlights the need for further investigation into the role of intratumoral fat deposition in CRLM prognosis.

Therefore, this study aimed to examine the frequency and clinical relevance of intratumoral fat deposition in colorectal liver metastases (CRLM) post-chemotherapy using dual-echo chemical shift gradient-echo magnetic resonance imaging (MRI).

## Material and methods

### Study population

Since our study was retrospective, informed consent was not obtained from members of the study group. The Maria Sklodowska-Curie National Research Institute of Oncology Ethics Committee in Warsaw, Poland, granted approval for this study. Our analysis adhered to the Strengthening the Reporting of Observational Studies in Epidemiology (STROBE) guidelines to enhance the study’s quality [9]. Our research project examined 98 successive patients with CRLM at a single medical institution (69 M, mean age 62.87 ± 10.73 years old undergoing MRI after chemotherapy. The study population was divided into two main comparison groups based on the presence of intratumoral fat deposition (FAT group vs. NO-FAT group) after neoadjuvant chemotherapy. The analysis comprised patients examined in the Department of Radiology, the Maria Sklodowska-Curie National Research Institute of Oncology in Warsaw, Poland, from January 2017 to December 2022. The decision to administer neoadjuvant chemotherapy for CRLM was made on a case-by-case basis, depending on several factors, including tumor resectability, patient performance status, and the extent of liver involvement. In our study, all patients received neoadjuvant chemotherapy, but the reasons for initiating chemotherapy varied based on initial resectability assessments at the discretion of the multidisciplinary team. Records were acquired from electronic health databases.

The inclusion criteria were as follows:

- Patients with histopathological confirmation of CRC and CRLM
- Undergoing MRI scans after preoperative chemotherapy
- Obtaining images of sufficient quality with minimal imaging and motion artifacts in the regions of interest

The exclusion criteria were as follows:

- Coexistence of more than one cancer at the same time
- Presence of liver metastases from cancers of unknown primary (CUP)
- Patients with confirmed CRC but without liver metastases
- Examinations performed on two different scanners (1.5 T and 3 T) of the patient
- Patients who had not undergone preoperative chemotherapy
- Death due to surgical complications or non-tumor-related complications
- Patients with CRLM too small for evaluation of intratumoral fat on MR images
- Contraindications to MR examination and intravenous administration of paramagnetic contrast agent:

- ○ Implanted pacemaker/cardioverter-defibrillator
- ○ Central nervous system vascular clips
- ○ Auditory implant
- ○ Insulin pumps
- ○ Metallic foreign bodies in the eyeball
- ○ Claustrophobia
- ○ A significant renal insufficiency (eGFR < 30 ml/min)
- ○ Transfer of the patient to another oncology center

The groups were matched based on the following characteristics to minimize potential confounding factors:

- **Age:** both groups included patients aged 30–75 years.
- **Chemotherapy regimen:** all patients in both groups received the same standard neoadjuvant chemotherapy regimen, which consisted of FOLFOX (5-fluorouracil, leucovorin, and oxaliplatin) or FOLFIRI (5-fluorouracil, leucovorin, and irinotecan), with or without targeted agents (bevacizumab or cetuximab).
- **Tumor burden:** the pre-treatment tumor burden was assessed using RECIST criteria, and the tumor size, number of metastases, and liver involvement were similar between the groups to ensure comparability.
- **Response to therapy:** both groups included patients who exhibited partial or complete radiological response after therapy, assessed by imaging before surgery.

### Imaging techniques

Twenty-six patients underwent MRI examinations on a 1.5-T scanner (Aera, Siemens), while 72 patients underwent imaging on a 3.0-T scanner (Signa Architect, GE, or Skyra, Siemens). The Dixon technique was used to perform axial T1-weighted imaging, ensuring uniform fat suppression in both in-phase and opposed-phase scans through chemical shift imaging [10, 11]. Table 1 provides an overview of the scanners and their scanning settings. Gadobenate dimeglumine (GBCA) (MultiHance®, Bracco Imaging, Germany), a liver-specific MR contrast agent, was given to all of the patients so that dynamic and hepatobiliary phases of contrast-enhanced MRI could be obtained. Axial and coronal T2-weighted, fat-suppressed T2-weighted, and DW images with multiple *b* values (0, 150, 500, 1000, 1500 s/mm^2^) were also acquired. We assessed the presence of fat accumulation within CRLM and examined the signal strength in the most concentrated fat regions. A drop in signal intensity of ≥ 12% from the in-phase image to the out-of-phase image showed that fat had been deposited inside the tumor. The data from the images was saved in PACS (Picture Archiving & Communication System, Centricity, GE HealthCare).

**Table 1:** Scanning parameters for dual-echo gradient-recalled echo MRI

Parameters	1.5-T scanner, *Aera*, *Siemens*		3-T scanner			
			*Skyra*, *Siemens*		*Signa Architect*, *GE*	
	In phase	Opposed phase	In phase	Opposed phase	In phase	Opposed phase
Repetition time, TR (ms)	120	120	150	150	130	130
Echo time, TE (ms)	4.8	2.4	4.8	2.4	4.8	2.2
Echo-train length (ETL)	1	1	8	8	12	12
Flip angle (°)	70	70	80	80	75	75
Slice spacing (mm)	2.5	6	0.5	0.5	0.5	0.5
Section thickness (mm)	4	4	1	1	4	4
Image matrix (%)	256 × 160	256 × 192	256 × 160	256 × 160	320 × 256	320 × 256
Field of view, FOV (mm)	300	300	400	400	350	350

### Imaging analysis

We assessed 98 MRI scans individually. The evaluation technique involved two seasoned radiologists independently reviewing the images on PACS, with 20 and 5 years of experience in abdominal MRI, respectively. The radiologists were aware of CRC as an underlying disease, but they were unaware of the patients’ personal data, the results of other imaging modalities, and the surgical or histopathological results after resection. Each liver segment was examined to document the quantity and dimensions of identified lesions, utilizing liver maps and adhering to the Couinaud classification [12]. The signal intensity index (SI) of CRLM was calculated using the formula shown below [13–17] as a way to measure the amount of fat inside the tumor:

$$\text{SI}\hspace{0.17em}=\hspace{0.17em}({\text{SI}}_{\text{IP}}-{\text{SI}}_{\text{OP}})/{\text{SI}}_{\text{IP}}\hspace{0.17em}\times \hspace{0.17em}100$$

where SI_IP_ and SI_OP_ represent the signal intensities of the lesion on in-phase and opposed-phase T1-weighted images, respectively. We determined the signal intensities of CRLM containing fat by drawing an elliptic region of interest (ROI) as large as possible (a minimum area of 12 mm^2^) to cover the densest region of fat deposition in CRLM in each patient. In the case of focal or heterogeneous intratumoral fat deposition, the ROI was drawn focally to cover only the region with fat deposition. To determine which lesion had the highest SI value, SI was measured for all lesions with intratumoral fat in each patient. SI_IP_ and SI_OP_ were measured three times, and the mean values were used for analysis. Lesions with evident artifacts (particularly lesions on the left hepatic lobe or in the hepatic dome) were excluded from evaluation. The effectiveness of chemotherapy was assessed by analyzing MRI results following RECIST 1.1 guidelines.

### Histopathological analysis

An expert pathologist specializing in gastrointestinal pathology (10 years of experience) performed all histological examinations independently of any clinical information. The primary colorectal tumors were categorized into six histopathologic types following the Japanese classification of colorectal carcinoma [18]. The pathological examination of surgically removed liver specimens confirmed the lesions. Tumor samples underwent hematoxylin and eosin (H&E) staining, followed by immunohistochemical staining using a panel of cytokeratin 7, cytokeratin 20, and CDX2 markers to confirm the intestinal origin of the lesions. In addition to H&E staining, Oil Red O staining was performed in selected cases to specifically highlight the presence of neutral lipids, further confirming the presence of fat within tumor cells or the surrounding stroma. The quantification of adipocytes was performed semi-quantitatively. For each tumor, fat content was evaluated across multiple histological sections to account for heterogeneity in fat distribution within the tumor. The presence of adipocytes was categorized based on the proportion of the tumor area containing fat cells, as follows:

- Focal accumulation: fat cells were present in small, localized clusters, generally accounting for less than 10% of the tumor area.
- Diffuse accumulation: fat cells were distributed more extensively throughout the tumor, involving more than 10% of the tumor area.

This semi-quantitative approach allowed us to classify tumors based on the extent of fat deposition, providing a framework to differentiate between minimal (focal) and significant (diffuse) fat accumulation [19].

### Statistical analysis

Predictors of intratumoral fat were determined through multivariate analyses. The presence of intratumoral fat deposition was linked to overall patient survival. Groups were compared based on their clinical features and results. Mean ± standard deviation or median (1st–3rd quartile) were used for continuous variables and absolute values and percentages for categorical variables. To verify normality, the Shapiro–Wilk test was employed, with subsequent group comparisons carried out using either Student’s two-sample *t*-tests or Mann–Whitney *U* tests, depending on the data distribution. The *χ*^2^ test was used to examine variations in proportions among different groups. The survival of patients was analyzed using the Kaplan–Meier technique, and the comparison of survival rates was done through the log-rank test. Survival time predictors, including the impact of intratumoral fat deposition, were identified through univariate and multivariate analysis using the Cox proportional hazards model. The results were expressed as hazard ratios (HR) and 95% confidence interval (CI). Statistical significance was attributed to tests where the *p*-value was equal to or less than 0.05. All analyses were performed using Jamovi version 2.5.5 software [20–28].

## Results

### Intratumoral fat before and after chemotherapy

**Fig. 1:**
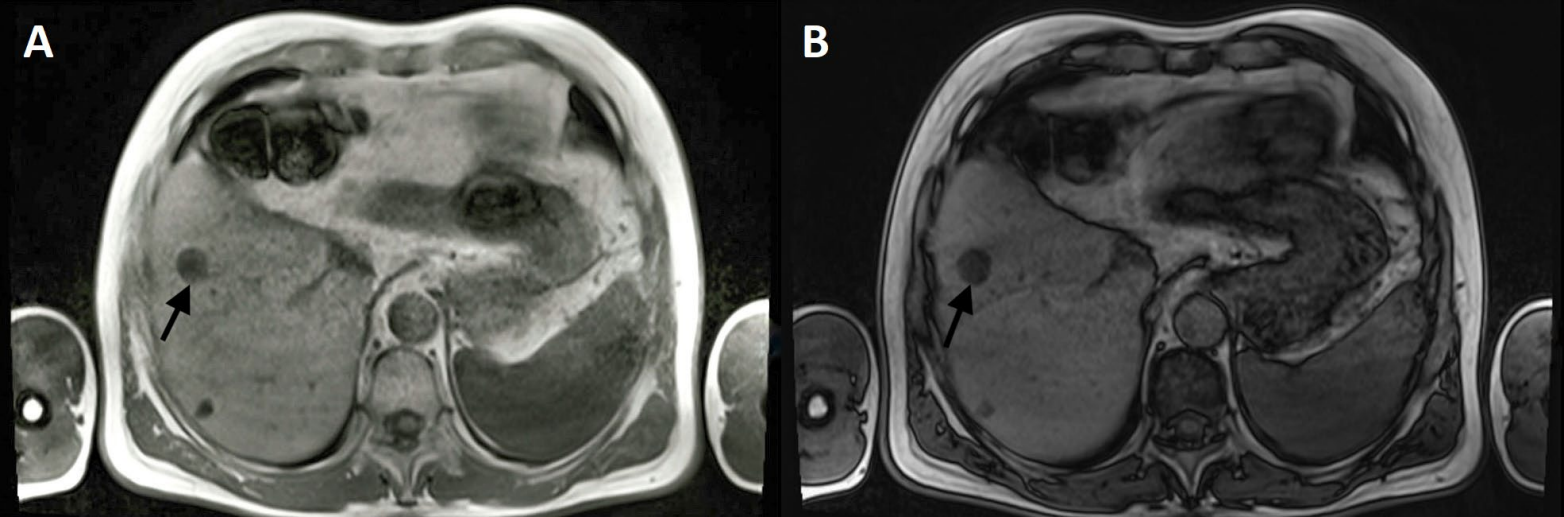
A 65-year-old man with CRLM after FOLFOX-4 chemotherapy in chemical shift MR imaging (arrows). Axial T1-weighted opposed-phase MR image shows focal signal intensity drop (**B**) compared to in-phase MR image (**A**)

Table 2 details the characteristics of the individuals included in this research (*n* = 98). In our study group, we defined 314 CRLM. Before chemotherapy, no patient (0%) exhibited intratumoral fat in CRLM. On MR images, intratumoral fat in CRLM was qualitatively detected in 29 of 98 (29.59%) patients after chemotherapy (Fig. 1). In 45 patients with intratumoral fat in CRLM, the fat deposition patterns were either focal (*n* = 11) or diffuse (*n* = 34). There was no statistical difference between intratumoral fat exhibition and the number of CRLM (Spearman rank correlation, *p* = 0.802). Twelve patients exhibited fat deposition in all depicted CRLM.

**Table 2:** Patient characteristics

Factor	FAT group, *n* = 29 No. (%) or mean ± SD or median	Non-FAT group, *n* = 69 No. (%) or mean ± SD or median	*p*-value
Age (years)	64.93 ± 9.11	62 ± 11.3	0.306
Sex (male), no. (%)	23 (79.31%)	46 (66.67%)	0.317
Site of primary tumor, no. (%)			
Right colon	1 (3.45%)	13 (18.84%)	0.062
Left colon	28 (96.55%)	56 (81.16%)	
Rectum	0 (0.00%)	12 (21.42%)	
Histopathologic types of primary tumors, no. (%)			
A well-differentiated adenocarcinoma	9 (31.03%)	23 (33.33%)	0.401
Moderately differentiated adenocarcinoma	17 (58.62%)	39 (56.52%)	
Mucinous adenocarcinoma	1 (3.44%)	4 (5.8%)	
Papillary adenocarcinoma	2 (6.90%)	1 (1.45%)	
Not available	0 (0.00%)	2 (2.9%)	
Dimension of CRLM (mm)	37.79 ± 27.48	30.39 ± 20.59	0.317
Number of CRLM lesions			
Median	5	4	0.802
Range	1–25	1–20	
Fluorouracil-based chemotherapy regimen, no. (%)			
Oxaliplatin	16 (55.17%)	38 (55.07%)	0.709
Irinotecan	8 (27.59%)	17 (24.64%)	
Oxaliplatin and irinotecan	3 (10.34%)	8 (11.59%)	
Neither oxaliplatin nor irinotecan	1 (3.45%)	1 (1.45%)	
Two or more regimens	1 (3.45%)	5 (7.25%)	
Bevacizumab, no. (%)			
Yes	5 (17.24%)	6 (8.7%)	0.198
No	24 (82.76%)	63 (91.3)	
Cetuximab or panitumumab, no. (%)			
Yes	6 (20.69%)	13 (18.84%)	0.720
No	23 (79.31%)	56 (81.16%)

### Overall survival

In our study, 54 (55.1%) of the patients were given FOLFOX-4 (folinic acid (leucovorin or calcium folinate)) along with 5-fluorouracil and oxaliplatin. Of these patients, 16 (29.63%) had fat inside the tumor. The FOLFIRI (folinic acid (leucovorin or calcium folinate) in conjunction with 5-fluorouracil and irinotecan) protocol represented the second group, with 25 (25.51%) patients; 8 (32.0%) of them presented with fat in CRLM. Of the 14 (14.29%) patients who received capecitabine, 5 (5.10%) had simultaneous treatment with oxaliplatin. Cetuximab and bevacizumab were most frequently administered together with oxaliplatin. There was no statistical difference between chemotherapy groups and patients’ overall survival (*p* = 0.709).

The median follow-up for the whole cohort was 32 months.

**Fig. 2:**
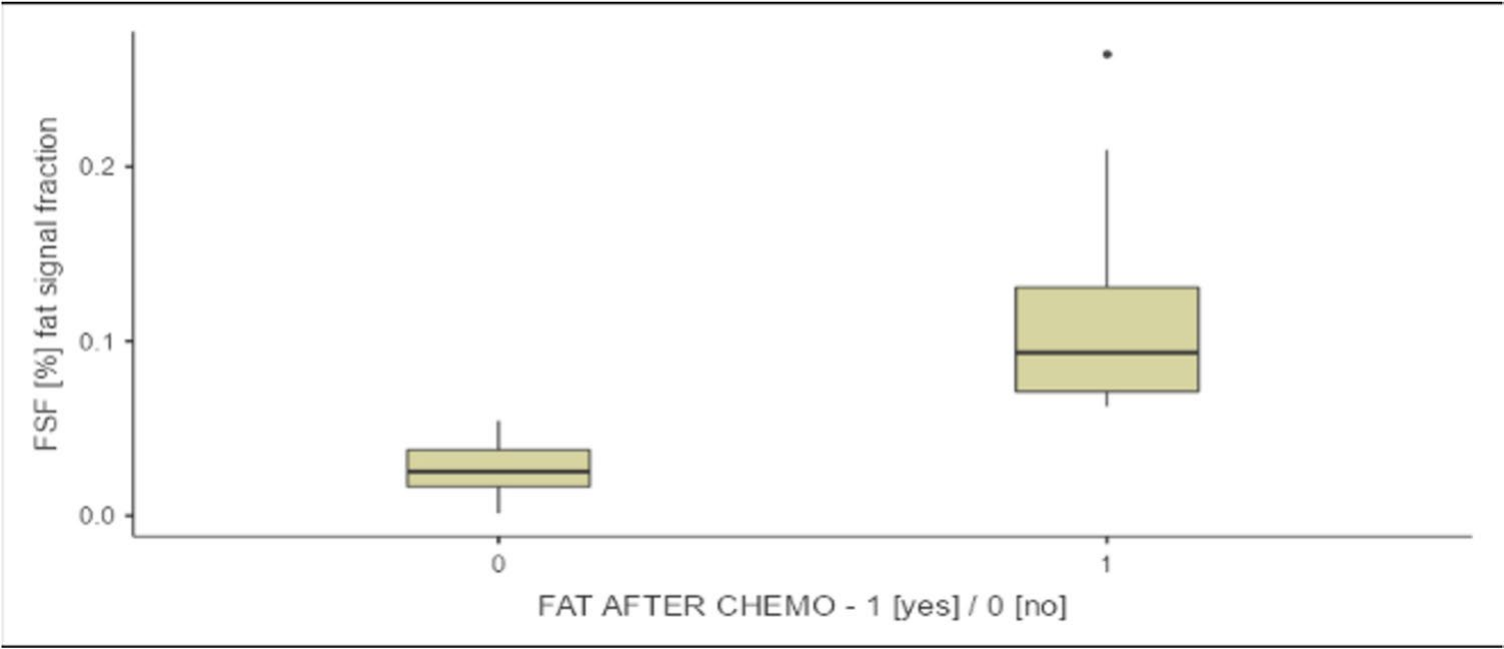
Fat signal fraction ≥ 12% and presence of fat after chemotherapy

There is a statistical difference between the fat group and no-fat group in survival time (Fig. 2).

**Fig. 3:**
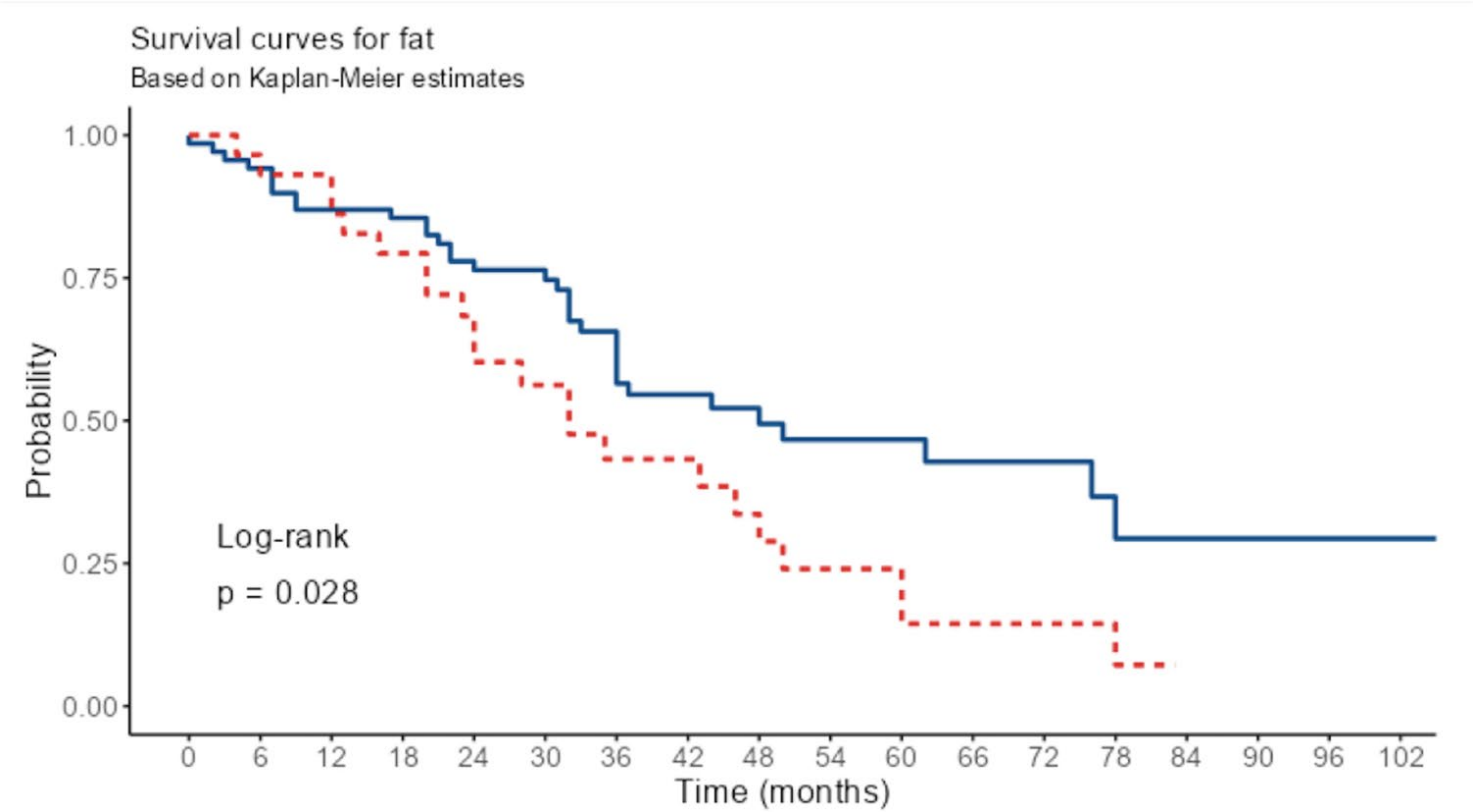
Multivariate survival analysis of patients with CRLM exhibiting intratumoral fat deposition

The outcomes of the Kaplan–Meier curve analysis demonstrated poorer overall survival among patients with intratumoral fat deposition (Fig. 3).

In patients with intratumoral fat deposition in CRLM, the overall survival was shorter (32 months (24–60, 95% CI)) compared to non-fat CRLM (48 months (36 NA, 95% CI)).

Table 3 shows how multiple factors like fat, tumor dimension, age, or signal intensity index affect survival. The multivariable-adjusted hazard ratios (HR) for tumor dimension and age are very close to 1. Only HR for fat and SI index are greater than 1. However, none of these parameters was statistically significant. Based on the univariate analysis, the overall mortality risk was greater for patients with intratumoral fat deposition in CRLM HR 1.81 (95% CI 1.06–3.10, *p* = 0.030).

**Table 3:** Multivariable-adjusted hazard ratio (HR) for intratumoral fat deposition in CRLM

Dependent		All	HR (univariable)	HR (multivariable)
FAT	0	69 (70.4)	-	-
	1	29 (29.6)	1.81 (1.06–3.10, *p* = 0.030)	2.11 (0.85–5.23, *p* = 0.106)
Dimension	Mean (SD)	32.6 (23.0)	1.00 (0.99–1.01, *p* = 0.851)	1.00 (0.98–1.01, *p* = 0.514)
AGE	Mean (SD)	62.9 (10.7)	0.99 (0.96–1.02, *p* = 0.411)	0.98 (0.96–1.01, *p* = 0.282)
SI	Mean (SD)	10.1 (9.4)	1.02 (1.00–1.05, *p* = 0.082)	1.79 (0.66–4.86, *p* = 0.254)

Fifty-six patients (57.14%) died during the study period. Of the 98 patients, 71 (72.44%) developed tumor recurrence (local or metastatic).

## Discussion

In our cohort, overall survival was lower in CRC patients with intratumoral fat deposition in CRLM after neoadjuvant chemotherapy. MRI emerges as a cornerstone in the surveillance of colorectal liver metastases, offering unparalleled sensitivity and specificity in lesion detection and characterization [29]. Due to its noninvasive nature, absence of ionizing radiation, and capacity to provide comprehensive anatomical and functional data, it is an essential instrument in treating individuals with CRC, ensuring straightforwardness and impartiality [29].

According to reports, the success of chemotherapy and the improved results post-CRLM resection are associated with the extent of fibrosis present in treated metastases, resulting in successful treatment [30]. In CRLM, there is typically central fibrosis or necrosis, with viable cells located at the outer edges. This results in chemotherapy-induced alterations being more noticeable in the peripheral regions [31]. Subasinghe et al. noted that CRLM manifests both usual-type necrosis (UN) and infarct-like necrosis (ILN). Simultaneously, ILN represents an uncommon type of cell death occurring in secondary liver tumors from colorectal cancer, typically emerging post-chemotherapy as a favorable reaction to chemotherapeutic agents [4, 32].

Several factors influence survival in CRLM, such as the progression-free interval after surgery for the primary tumor, whether the tumor is metachronous or synchronous, KRAS or BRAF mutation status, CEA levels, and extrahepatic disease [33]. Prior research indicates a potential connection between intratumoral fat deposition and the effectiveness of chemotherapy on CRLM. However, the relationship between intratumoral fat deposition and prognosis remains an area of ongoing research [34]. Survival rates of individuals with CRLM that included fat were shown to be shorter by Nakai et al. in comparison to patients with non-fat CRLM in a group of 59 individuals [8]. Similar to previous reports, the Kaplan–Meier curve analysis in our group also indicated poorer overall survival among patients with tumor SI values of 12% or more. The accuracy of our research was greater than what has been recently reported. We prepared our study on a larger sample size with a longer observation time, proving that the presence of intratumoral fat after chemotherapy negatively influences the long-term prognosis.

Recent literature shows that median overall survival for patients with CRLM who undergo surgical resection is now reported to range from 30 to 50 months, depending on factors such as tumor biology or treatment strategies. For patients receiving chemotherapy and targeted therapies without surgery, median overall survival can range from 18 to 36 months, with variations based on the effectiveness of treatment and individual tumor response [35]. The 5-year survival rate increases to 50% in patients deemed suitable for receiving both chemotherapy and surgery, particularly before the operation, which was almost unheard of in earlier decades [32–35]. Overall survival rates were very different in our group depending on whether a patient had intratumoral fat in CRLM or not. In practice, patients without fat lived longer with the same chemotherapy protocols. However, we assumed that different chemotherapy protocols might influence the overall survival of patients. Zhou et al. highlighted multiple possible responses to the treatment and molecular mechanisms of cancer microenvironment evolution after chemotherapy, which might impact our results [36]. Fatty liver is the factor that results from microenvironment disturbances and influences current results, as mentioned in previous studies. However, our study did not assess factors associated with fatty liver [37]. Hepatic steatosis has an unclear effect, but authors strongly believed that fatty change in the liver has an impact on the development of CRLM [38–40].

The limitations of the study include a restricted number of eligible patients who received fluorouracil-based chemotherapy regimens, as well as the use of different MRI scanners (1.5 T/3 T); however, all images were acquired using the same principal sequence. Additionally, the individual factors affecting patient survival in CRLM, as discussed earlier, could potentially introduce bias into our statistical analyses. Therefore, it is essential to include the mentioned survival risk factors to better understand the true impact of lipid retention. Another limitation of the study is the lack of correlation between chemical shift imaging in MRI with histopathological results due to the fact that all of the patients were undergoing chemotherapy, which may have influenced the results. To increase generalizability, further studies comprising larger patient groups and different medical centers are required to broaden the cross-sectional knowledge of intratumoral fat deposition in CRLM after chemotherapy in multiple oncological centers. Nevertheless, the detection of intratumoral fat in CRLM enriches the prospective evaluation in terms of prognostic value and the impact of our results on therapeutic decisions. It offers the potential for more effective diagnoses and more personalized therapeutic approaches.

## Conclusion

In our group, nearly 30% of CRLM patients exhibited intratumoral fat after chemotherapy. Patients with intratumoral fat deposition in CRLM have a shorter overall survival time. The presence of fat in CRLM correlates with a poor long-term prognosis. MRI is sensitive to detecting fat changes after neoadjuvant chemotherapy, but more rigorous validation is needed. Future studies should focus on optimizing the cut-off for fat detection, potentially comparing MRI results with histopathological findings in larger, well-characterized cohorts.

## Data Availability

No datasets were generated or analyzed during the current study.
